# Performance-Based and Self-Reported Frailty in Older Adults with or Without Fibromyalgia

**DOI:** 10.3390/ejihpe16030036

**Published:** 2026-03-04

**Authors:** Dylan G. Serpas, Jordan K. Aquino, Laura Zettel-Watson, Barbara J. Cherry

**Affiliations:** 1 Department of Psychology, California State University, Fullerton, CA 92831, USA; 2 Department of Psychology, University of South Florida, Tampa, FL 33620, USA; 3 Department of Public Health, California State University, Fullerton, CA 92834, USA; 4 Department of Behavioral & Community Health, University of Maryland School of Public Health, College Park, MD 20742, USA; 5 Aging Studies Program, California State University, Fullerton, CA 92831, USA

**Keywords:** fibromyalgia, chronic pain, frailty, aging, principal component analysis

## Abstract

Background: Fibromyalgia (FM) is a chronic widespread pain condition implicated in accelerated aging, functional decline, and physical frailty. Objective: This study examined differences in performance-based and self-reported physical frailty phenotypes among middle-aged and older adults with and without FM. Materials and Methods: A cross-sectional sample of 234 community-dwelling middle-aged and older adults with (59.0%) or without FM was analyzed. Physical frailty was defined as weakness, low physical activity, exhaustion, and slowness, assessed using validated performance-based (Fullerton Advanced Balance Scale [FAB], 8-foot up and go test [8FUPGT], 30-second chair stand [30SCS], 6-minute walk [6MWT], 30-foot walk [30FW]) and self-report measures (Rapid Assessment of Physical Activity [RAPA], fatigue numeric rating scale). Principal component analysis (PCA) evaluated the underlying structure of physical frailty indicators, yielding performance-based and self-reported components. Standardized factor scores were used as outcomes in regression analyses examining associations with pain intensity. Results: PCA supported a two-component frailty structure explaining 61% of the variance. After adjusting for age, gender, depressive symptoms, and body mass index, greater pain intensity was associated with worse performance-based (B = −0.10, *p* < 0.001; adjusted R^2^ = 0.36) and self-reported (B = −0.10, *p* < 0.001; adjusted R^2^ = 0.39) frailty. Discussion: Findings suggest that pain intensity is associated with frailty risk among aging adults, supporting the clinical utility of both performance-based and self-reported physical frailty assessments in FM.

## 1. Introduction

Frailty is conceptualized as a gradual and cumulative decline in normative functioning across the lifespan (Clegg et al., 2013). Frailty—a metric representing population aging—is not a discrete event and, instead, signifies a process of increased vulnerability through declining functional capacities (Clegg et al., 2013). Findings from nationally representative data show that frailty is positively associated with the prevalence of chronic disease and disability among older adults (Bandeen-Roche et al., 2015). Additionally, frailty among aging adults poses a public health concern, as pre-frail and frail aging adults demonstrate a greater risk of falls than their non-frail counterparts (Cheng & Chang, 2017; Ensrud et al., 2008; Samper-Ternent et al., 2012) and approximately half of older adults with frailty report experiencing a fall in the past year (Bandeen-Roche et al., 2015). As falls represent the leading cause of accidental death among adults aged 65 and older (Burns & Kakara, 2018), the identification of effective strategies to detect frailty and mitigate the risk of disability and mortality among aging adults is paramount.

The prevalence of frailty varies depending on the method of assessment. A recent systematic review and meta-analysis revealed that, across 62 countries and territories, the pooled prevalence of physical frailty was 12% in nationally representative samples (O’Caoimh et al., 2021). O’Caoimh et al. (2021) also reported that rates of physical frailty increased with age such that the prevalence for studies including participants aged 50+ and 90+ was 11% and 51%, respectively. Evidence also indicates that physical frailty is more common among women (Collard et al., 2012). There is a surplus of instruments designed to measure frailty with a recent systematic review identifying 51 published frailty measures that generally offer a consensus on the definition of the construct (Faller et al., 2019). Of note, Faller et al. (2019) identified robust heterogeneity in the interpretation and psychometric properties across the 51 frailty instruments (Faller et al., 2019). Generally, most frailty instruments measured physical phenotypes including slowness, weakness, or exhaustion (Faller et al., 2019). This study focuses on physical phenotypes of frailty.

Several correlates of physical frailty have been identified, including age, insomnia, mood disorders, cognitive impairment, and pain (Collard et al., 2012; Liu et al., 2021). Indeed, a systematic review of chronic pain and physical frailty among community-dwelling older adults found that, across 23 studies, the prevalence of chronic pain among frail older adults was about 45%, with some estimates as high as 70% (Reyes et al., 2019). Separate lines of literature propose pain to be either a risk factor (Bindawas et al., 2018; Dapp et al., 2014; Megale et al., 2018; Rodriguez-Sanchez et al., 2019; Sodhi et al., 2020; Veronese et al., 2017; Wade et al., 2016, 2017) or a consequence of physical frailty (Guerriero & Reid, 2020; Karp et al., 2008; Lohman et al., 2017; Oyon et al., 2022; Shega et al., 2012). In support of the conjecture that physical frailty is a consequence of pain, a recent systematic review and meta-analysis of 24 studies found that individuals with chronic pain were approximately twice as likely as their pain-free counterparts to develop physical frailty after an average follow-up of approximately six years (Lin et al., 2020). Similarly, Saraiva et al. (2018) systematically reviewed and meta-analyzed five prospective longitudinal studies (two measured physical frailty) on persistent pain and frailty and found that individuals with persistent pain at baseline were twice as likely to develop frailty at follow-up (which ranged from 3 to 8 years). Saraiva et al. (2018) noted that two of the five studies did not adjust for key confounds relevant to the persistent pain and frailty association that could explain differences in frailty trajectories (e.g., type of chronic pain). Though scholars have discussed potential shared mechanisms in physical frailty among localized and widespread chronic pain conditions (Staud, 2011), aging adults with localized chronic pain may present different patterns of frailty than aging adults with chronic widespread pain. Some research has supported pain as a predictor of global frailty and has included participants with chronic widespread pain (e.g., Wade et al., 2016). However, most studies examining pain and physical frailty among aging adults have excluded individuals with chronic widespread pain (Lin et al., 2020; Saraiva et al., 2018), including conditions characterized by chronic widespread pain such as fibromyalgia (FM).

FM is a condition characterized by chronic widespread pain and is frequently accompanied by co-occurring conditions including mood disorders, fatigue, and poor sleep quality (Kleykamp et al., 2021; Wolfe et al., 2010). FM affects 2–5% of the United States population (G. T. Jones et al., 2015; Wolfe et al., 2010). Rates of FM are higher among women (Ursini et al., 2021; Wolfe et al., 2018) and individuals with autoimmune rheumatic diseases (Fitzcharles et al., 2018; Wolfe et al., 2018) and obesity (D’Onghia et al., 2021). FM may result in diminished quality of life, poor occupational productivity (Arnold et al., 2008; McDonald et al., 2011), and high economic costs (D’Onghia et al., 2022). Given the unique symptom profile of FM, individuals with FM may be at an increased risk of physical frailty compared to those with other chronic pain conditions or individuals without pain. For example, Lieber et al. (2022) examined the prevalence of physical frailty among adults aged 65 and older with one of three types of inflammatory arthritis (rheumatoid arthritis, ankylosing spondylitis, psoriatic arthritis) and found that the prevalence of FM was greater among frail as compared to non-frail participants across all rheumatic conditions. In addition, the lifetime prevalence of osteoarthritis in FM is approximately 40% (Kleykamp et al., 2021), and osteoarthritis is a risk factor for physical frailty (Salaffi et al., 2021). Recently, scholars have urged for the incorporation of pain into the construct of frailty to improve clinical assessment and care (Guerriero & Reid, 2020). Researchers have also advocated for frailty to be integrated into the conceptualization of FM (Martínez-Velilla & Fernández-Solà, 2015), based on evidence demonstrating markers of premature aging in FM including accelerated age-related gray-matter changes in the brain (Kuchinad et al., 2007) and shortened telomere length (Hassett et al., 2012). Thus, it is important to consider chronic pain conditions such as FM as a possible mechanism in advanced or early onset physical frailty.

Limited research has empirically examined physical frailty in FM. Nonetheless, studies have found that adults with FM report poorer scores and performance on indicators of physical frailty, including weakness, low physical activity, exhaustion, and slowness. For instance, C. J. Jones et al. (2015) found that women with FM performed below criterion fitness standards on physical performance assessments of strength, agility, and balance. Physical activity was positively correlated with physical performance scores, whereas pain intensity and depressive symptoms were negatively correlated (C. J. Jones et al., 2015). Studies have also shown that individuals with FM demonstrate hallmarks of physical frailty such as poor physical performance on assessments measuring balance performance (Núñez-Fuentes et al., 2021; Serpas et al., 2023; Vaillant et al., 2016), low physical activity (Cherry et al., 2014), weakness (Serpas et al., 2023), and exhaustion (Cherry et al., 2014; Serpas et al., 2023) and report more symptoms of normative aging such as fatigue (Serpas et al., 2023) compared to non-FM controls. Taken together, there is evidence to suggest that individuals with FM evince indicators of physical frailty consistent with premature aging. However, the construct of physical frailty remains understudied among aging adults with FM.

The purpose of this study was to (a) assess differences in performance-based and self-reported physical frailty between middle-aged and older adults with and without FM and (b) examine whether the number of years of symptoms of FM is associated with performance-based and self-reported physical frailty. We proposed the following hypotheses: (1) Middle-aged and older adults with more versus less pain (FM vs. non-FM) will demonstrate poorer performance-based and self-reported physical frailty and (2) performance-based and self-reported physical frailty will vary by time since FM symptom onset among individuals with FM.

## 2. Materials and Methods

### 2.1. Eligibility Criteria

To enroll in this study, participants were required to be 50 years of age or older, community-dwelling, and functionally independent. Participants could not enroll in the study if they were unable to independently walk for at least 6 min or reported a pre-existing medical condition that would compromise their safety during moderate exercise.

### 2.2. Participants

Participants (n = 234) in this cross-sectional study were drawn from six waves of a longitudinal study among community-dwelling adults with and without FM. Participants were recruited from 2008 to 2018 every two years. Data were combined across waves for participants’ entry (first assessment) into the study. The sample comprised community-dwelling aging adults with FM (55.3%) or without whose ages ranged from 50 to 87 (M = 63.79, SD = 8.72). Participants were largely racially white (89.1%), women (83.3%), married (61.2%), retired (44.8%), and possessed a professional or graduate degree (33.3%). Sociodemographic characteristics of the study sample are provided in Table 1.

### 2.3. Study Design and Procedure

A non-randomized, purposive sampling method was used to recruit the study population for this cross-sectional, observational study. Enrolled participants provided informed consent and a packet containing sociodemographic questionnaires was mailed to them before scheduling an appointment for in-person data collection. During in-person data collection, the Rapid Assessment of Physical Activity (RAPA) was completed with a trained interviewer. The Beck Depression Inventory-II was also administered at this time. Physical performance tasks were subsequently administered individually in private spaces by trained research assistants. Participants were screened for cognitive impairment using the Mini-Mental Status Examination (MMSE; Folstein et al., 1975), a measure of cognitive capacities including language, visual–spatial skills, attention, orientation, and memory. MMSE scores of 24 or lower suggest cognitive impairment (Folstein et al., 1975). Participants scoring 24 or below were excluded from analysis (n = 6).

### 2.4. Instruments

Sociodemographic characteristics: Participants provided information regarding age, gender, income, education, employment status, height, weight, and FM characteristics including onset and pain intensity (0 = no pain, 10 = worst pain).

Depressive symptoms: Depressive symptoms were assessed using the Beck Depression Inventory-II (BDI-II), a 21-item self-report measure of depressive symptomatology over the past two weeks. Each item is rated on a 4-point Likert scale ranging from 0 to 3, with higher total scores indicating greater depressive symptom severity. The BDI-II has demonstrated strong psychometric properties among aging adults with FM (Serpas et al., 2022). In the current study, the internal consistency reliability for the BDI-II was strong (α = 0.881).

Frailty: Frailty is posited to comprise five domains: Weakness, low physical activity, exhaustion, slowness, and weight loss (Fried et al., 2001; Xue, 2011). Xue (2011) conceptualized frailty solely through physical phenotypes, which is a common practice based on existing measures of frailty (Faller et al., 2019). We recognize that broader conceptualizations of frailty exist (Faller et al., 2019); however, physical manifestations were a focus of this study to examine indicators of physical deterioration correlated with chronic widespread pain in FM as an initial step in studying the expression of frailty in this population. Categorical cutoffs were not utilized given evidence suggesting that clinical cutoffs with frailty measures may reduce precision because they do not provide predictions of outcomes as accurately as dimensional measures (Kim et al., 2022; Wu et al., 2018). In this study, weight was excluded due to the limited value in comparing self-reported to objective measures of weight in the context of frailty. Instead, BMI was included as a covariate in analyses. Physical frailty domains were operationalized with objective and self-report measures. Weakness was operationalized using the objective 30-second chair stand test and self-reported Rapid Assessment of Physical Activity 2. Low physical activity was operationalized using the objective Fullerton Advanced Balance and 8-foot up and go tests and the self-reported Rapid Assessment of Physical Activity 1. Low physical activity was operationalized using balance and mobility tasks, consistent with the physical frailty phenotype framework (Fried et al., 2001; Xue, 2011) in which reduced habitual activity manifests as impaired gait speed, balance, and movement performance. Lastly, exhaustion was operationalized using self-reported fatigue. Lastly, slowness was operationalized using the objective 6-minute walk and 30-foot walk tests.

The 8-foot up and go test (8FUPGT; Rikli & Jones, 2013) was used to assess the low physical activity domain of frailty through static and dynamic postural control. While seated, participants were instructed to stand and walk as quickly and safely as possible to and around a cone placed 8 feet away and return to their seat. Two trials were administered and the fastest time in seconds was recorded where higher scores indicate poorer performance (Rikli & Jones, 2013). The 8FUPGT has demonstrated strong psychometrics with middle-aged and older adults with and without FM (Follick et al., 2016; Serpas et al., 2023).

Weakness: The 30-second chair stand (30SCS; Rikli & Jones, 2013) assessed the weakness domain of frailty through lower-body strength. Once seated, participants were instructed to stand up and sit back down as many times as they could within 30 s. More stands reflect better performance (Rikli & Jones, 2013). Previous studies have reported strong psychometric properties of the 30SCS among individuals with and without FM (Cherry et al., 2014; Serpas et al., 2022, 2023).

Balance: The Fullerton Advanced Balance (FAB; Rose et al., 2006) was used to measure balance. The FAB contains 10 items rated by a trained research assistant. Each item assesses different capacities in postural control. Items are rated on a 5-point ordinal scale (0 = unable to complete task, 4 = independent task completion) where higher scores indicate better balance performance (Rose et al., 2006). Item scores are summed with possible scores ranging from 0 to 40. The FAB is psychometrically equivalent to other objective balance assessments such as the Berg Balance Scale (Klein et al., 2011; Rose et al., 2006).

Slowness: The 6-minute walk test (6MWT; Rikli & Jones, 2013) measured the slowness dimension domain of frailty through cardiorespiratory fitness. Participants were instructed to walk along a flat 50-yard rectangular space as quickly and safely as possible. The maximum distance participants walked in yards was recorded. Higher scores indicated better performance (Rikli & Jones, 2013). The 6MWT has shown strong psychometric properties among middle-aged and older adults with and without FM (Follick et al., 2016).

The 30-foot walk (30FW; Theou et al., 2006) test assessed the slowness domain of frailty. The 30FW is an objective assessment of gait or cadence velocity that is negatively correlated with age (Tudor-Locke et al., 2012). To complete the 30FW, participants walked 30 feet as quickly and safely as possible. Time (seconds) and number of steps (feet) required to complete the distance were recorded. Cadence velocity was computed by dividing 30 feet by the time (seconds) to walk the distance, where higher scores indicate poorer performance.

Physical activity: The Rapid Assessment of Physical Activity (RAPA; Topolski et al., 2006) questionnaire was used to measure physical activity level. The RAPA is a self-report measure of physical activity among older adults. Part 1 (RAPA 1) consists of seven questions (yes/no) measuring level of aerobic physical activity. Participants indicated whether they engage in sedentary behavior or light, moderate, and vigorous physical activities with examples provided (e.g., light: I do some light physical activity every week). Participants are classified as either sedentary, underactive, underactive-light, underactive-regular, or active (Topolski et al., 2006). For the purpose of this study, RAPA 1 was treated as an interval variable for analysis (1 = sedentary, 5 = active) with higher scores indicating greater aerobic physical activity (RAPA 1). RAPA 1 was used to capture the low physical activity domain of frailty. Part 2 of the RAPA (RAPA 2) consists of two questions measuring strength and flexibility. Participants indicate whether they engage in activities to increase muscle strength, flexibility, or none. Participants were scored as participating in none, flexibility or strength activities, or both strength and flexibility. RAPA 2 was treated as an interval variable (1 = none, 2 = flexibility/strength, 3 = strength and flexibility) where higher scores indicated engaging in more strength and flexibility activities. RAPA 2 captured the weakness domain of frailty.

Fatigue: Fatigue was self-reported to assess the exhaustion domain of frailty. Fatigue was measured using the following question: “What was your average daily fatigue in the past week?” rated on an 11-point numeric rating scale (0 = no fatigue, 10 = worst fatigue). This item was drawn from the National Fibromyalgia Association Questionnaire (NFAQ; Bennett et al., 2007).

### 2.5. Analysis Plan

Descriptive statistics using means, standard deviations, frequencies, and proportions were calculated for all variables in SPSS version 31. Data were screened for univariate and multivariate normality assumptions. Variables showed minimal univariate skewness and kurtosis. Four multivariate outliers (Mahalanobis distance > 27.88, *p* < 0.001) were removed for analysis (n = 234).

To answer the proposed research questions, frailty was conceptualized based on past research (Xue, 2011). Domains of physical frailty used in this study included weakness, low physical activity, exhaustion, and slowness. Each domain was operationalized using either performance-based (i.e., 30SCS, FAB, 8FUPGT, 6MWT, and 30FW) or self-report measures (i.e., RAPA 1, RAPA 2, fatigue). Principal component analysis (PCA) was conducted to assess the correlational organization of the proposed frailty items. Identifying underlying latent constructs was not the objective of this study. Therefore, exploratory factor analysis was not an appropriate choice. Assessing the organization of the frailty variables and creating composites to answer the proposed research questions were of primary interest.

Variables contained 3% or less of missing data. Eleven participants contained missing data across any variable used in analysis. Little’s missing completely at random test was statistically significant, χ^2^(121) = 183.24, *p* < 0.001, suggesting that participants with complete data were distinguishable from those with incomplete data (Tabachnick & Fidell, 2019).

Little’s missing completely at random (MCAR) test indicated a statistically significant departure from complete randomness in the missing data, χ^2^(184) = 516.43, *p* < 0.001. The small number of missing cases precluded separate variance *t*-tests, which is expected when missingness is under 5%. Because MCAR is not a required assumption for factor analytic models and missingness was minimal, models were estimated using listwise deletion, which is appropriate for conditions where data are missing at random. Moreover, binary logistic regression analysis predicting data missingness showed that gender, age, depressive symptoms, 6MWT, FAB, 30SCS, 8FUPGT, RAPA 1, RAPA 2, 30FW, pain intensity, and fatigue were not significantly associated with data missingness (*p*’s > 0.05), suggesting that data could be missing at random. In addition, the proposed regression analyses for this study were performed with and without a missing data variable (1 = missing data, 0 = no missing data) as a predictor. The missing data predictor was not statistically significant (*p* > 0.05) across all regression models and its inclusion did not change the results. These observations coupled with the small proportion of missing data (≤3%) led us to utilize listwise deletion for analysis. Thus, the final analytic sample included 234 participants.

PCA was conducted on eight frailty variables using principal component factoring with oblique rotation (direct oblimin) to allow the factors to correlate as they would naturally (Fabrigar et al., 1999) and aid in component interpretation. Oblique rotation was selected because items and components were expected to be correlated. Oblique rotation is recommended when components share a correlation of 0.32 or greater (Tabachnick & Fidell, 2019). Of note, when inspecting component solutions with PCA, it is ideal for the model to demonstrate a simple structure where variables correlate highly with only one factor (Tabachnick & Fidell, 2019). The PCA solution was evaluated using the Kaiser–Meyer–Olkin (KMO) test to assess sampling adequacy where values above 0.60 are considered acceptable (Kaiser, 1970, 1974). Bartlett’s test of sphericity assessed the degree of interrelatedness among the variables (Bartlett, 1954). Bartlett’s test of sphericity tests the null hypothesis that the variables included in the PCA represent an identity matrix and are unrelated where *p* < 0.05 rejects the null hypothesis. The scree plot was inspected to determine the ideal number of factors considering the Kaiser–Guttman rule, which recommends retaining factors with eigenvalues greater than 1.0 (Kaiser, 1960).

Following PCA, standardized component scores were computed for each frailty domain. Subsequently, two multiple linear regression models were estimated using ordinary least squares. Models were performed to examine associations between pain intensity and objective and self-reported frailty standardized component scores, adjusting for age, gender, depressive symptoms, and BMI. Pain intensity was used as the predictor variable rather than FM diagnosis to capture variability in a primary clinical feature of FM. FM is a heterogeneous condition characterized by multiple symptoms, several of which overlap with indicators of physical frailty (fatigue, depressive symptoms, low physical activity). Thus, modeling pain intensity dimensionally allowed for a more precise test of associations while avoiding conflation of overlapping symptom domains with physical frailty. Moreover, pain intensity in FM has been documented to be more severe than that in other chronic widespread pain conditions (Reyes et al., 2019).

Two subsequent models were estimated among FM participants only to examine the effects of time since FM symptom onset on objective and self-reported standardized component scores, adjusting for age, gender, depressive symptoms, BMI, and pain intensity. Model assumptions were confirmed prior to analyses. FM symptom onset, instead of time since diagnosis, was used given the documented lag time between FM symptom onset and diagnosis (Choy et al., 2010). Regression coefficients are reported as unstandardized estimates (B), reflecting the expected change in standard deviation units of the standardized physical frailty factor scores for each one-unit increase in pain intensity (rated 0–10). Across all regression models, multicollinearity was evaluated by inspecting tolerance and variance inflation values (VIF). Values of tolerance above the recommended minimum of 0.10 and VIF below the threshold of 10 indicate no evidence of problematic multicollinearity (Tabachnick & Fidell, 2019).

## 3. Results

### 3.1. Preliminary Analyses

Gender differences in the frailty items were assessed, given prior reports of differences (Serpas et al., 2023). First, PCAs were conducted with and without men (n = 39) and appreciable differences were not observed. Some gender differences were found across frailty and related measures. Differences in frailty variables across sociodemographic characteristics were examined to identify possible covariates. Women reported significantly greater fatigue, t(232) = 4.04, *p* < 0.001, and lower 6MWT scores, t(232) = −2.31, *p* = 0.022. No other gender differences in frailty variables were found (*p* > 0.05). Gender was included as a covariate in regression analysis. Race/ethnicity was screened as a covariate. Due to small cell sizes, white and non-white participants were compared across all frailty variables. Independent samples *t*-tests identified no significant group differences across all frailty variables (*p* > 0.05). Spearman correlations indicated that income and level of education were not associated with any frailty variable (*p* > 0.05). A significant monotonic association was found between income and physical frailty variables; however, follow-up ANOVAs did not detect significant mean differences across income categories. Income was not retained as a covariate. Participants with FM reported significantly greater pain intensity than participants without FM, t(232) = −13.15, *p* < 0.001. This finding verified that pain intensity meaningfully differentiated participants by FM status, supporting its use as a proxy for FM diagnosis in subsequent analyses.

Bivariate associations of the study variables are provided in Table 2. Age was significantly associated with frailty variables and was, therefore, included as a covariate in all regression analyses. Given the statistically significant association between physical performance and depression in FM in prior research (C. J. Jones et al., 2015; Serpas et al., 2023; Soriano-Maldonado et al., 2016) and in the current study, depressive symptoms were included as a covariate. In addition, since weight was excluded due to the limited value in comparing self-report to objective measures of weight in the context of frailty, BMI was included as a covariate in regression analyses. An a priori linear regression power analysis using G*Power version 3.1.9.7 determined a minimum required sample size of 134 with 95% power based on a small to medium effect size for the proposed associations (Saraiva et al., 2018; Lin et al., 2020). Thus, a priori estimates indicated the proposed analyses were adequately powered. Lastly, multicollinearity diagnostics indicated no concerns across any regression model, given that tolerance values were well above the recommended minimum of 0.10 and VIF values were well below the threshold of 10 (Tabachnick & Fidell, 2019).

### 3.2. Principal Component Analysis

Initial inspection of intervariable correlations showed that the variables generally shared approximately 10% of variance, consistent with statistical recommendations (Tabachnick & Fidell, 2019; see Table 3). Results indicated that the KMO measure of sampling adequacy was acceptable (KMO = 0.83), and Bartlett’s test of sphericity was statistically significant, χ^2^(28) = 618.25, *p* < 0.001, indicating that, overall, correlations between the variables were non-zero and PCA may help inform the number of dimensions ideal for this model. Inspection of the scree plot showed a pronounced inflection point at the second highest eigenvalue. The analysis yielded an ideal two-component solution with eigenvalues greater than 1.0 explaining a total estimated 61% of the variance for the entire set of variables. Within the communalities, all the items were 0.38 and higher (h^2^ range: 0.38–0.74), indicating each variable shared some common variance with other variables in the model. The two components shared a correlation of 0.41, supporting the appropriateness of oblique rotation in model specification. Results of the PCA are provided in Table 3.

The pattern matrix indicated simple structure. Rotated component 1 was labeled as performance-based frailty due to high loading by the following items: FAB, 6MWT, 30SCS, 8FUPGT, and 30FW based on the pattern matrix (see Table 3). The first extracted component explained approximately 45% of the total variance. Rotated component 2 was labeled as self-reported frailty due to high loadings by the following items: RAPA 1, RAPA 2, and fatigue. The second component score explained approximately 15% of the total variability.

### 3.3. Differences in Frailty by Pain Intensity

Standardized component scores were computed for each extracted component and used as criteria for regression. Two multiple linear regression models were performed with each component score regressed on pain intensity, adjusting for age, gender, BMI, and depressive symptoms. When holding covariates constant, greater pain intensity was associated with worse performance-based frailty (B = −0.10, *p* < 0.001), indicated by higher 8FUPGT performance and lower 6MWT, 30FW, FAB, and 30SCS performance, adjusted R^2^ = 0.36. In the second model, greater pain intensity was associated with worse self-reported frailty (B = −0.09, *p* < 0.001), after adjusting for covariates, adjusted R^2^ = 0.39.

### 3.4. Length of Time with FM Symptoms and Frailty

Among FM participants, associations between the length of time with FM symptoms and each frailty component were assessed, adjusting for age, gender, depressive symptoms, BMI, and pain intensity. Length of time with FM symptoms was not associated with performance-based frailty (B = −0.04, *p* = 0.667), adjusted R^2^ = 0.34, or self-reported frailty (B = 0.02, *p* = 0.842), adjusted R^2^ = 0.14, after controlling for covariates. A post hoc power analysis based on a small effect size (R^2^ = 0.14) and 138 participants indicated 94% power was obtained.

## 4. Discussion

In the current study, we investigated domains of objective and self-report frailty, consistent with common conceptualizations of frailty (Fried et al., 2001; Xue, 2011). We predicted that FM participants would demonstrate poorer performance-based and self-reported frailty. Following PCA, pain intensity, a proxy for FM status, was significantly negatively associated with both objective-based and self-reported frailty measures. Of note, pain intensity was modeled as an indicator of current symptom severity instead of a substitute for FM diagnosis. This analytic decision was intended to minimize conceptual overlap between FM status and physical frailty indicators, several of which may be influenced by pain-related functional limitations. Importantly, pain severity was not used as a proxy for FM diagnosis but to examine whether current pain intensity, independent of categorical status, was associated with physical frailty.

To date, few studies have examined frailty in aging adults with FM. Existing research has found that FM is more common among physically frail compared to non-physically frail aging adults across several rheumatic conditions (Lieber et al., 2022; Wysham et al., 2025), suggesting that FM may play a role in the onset and progression of physical frailty. Moreover, frailty has been argued as an important element in the conceptualization of FM (Martínez-Velilla & Fernández-Solà, 2015), yet empirical progress in the assessment and identification of frailty in FM is minimal, despite a higher degree of pain behavior and pain interference in frailty (Lieber et al., 2022). Nonetheless, previous research has found that aging adults with FM demonstrate phenotypes consistent with physical frailty (Fried et al., 2001; Xue, 2011), including poor balance (Núñez-Fuentes et al., 2021; Serpas et al., 2023; Vaillant et al., 2016), low physical activity (Cherry et al., 2014), weakness (Serpas et al., 2023), exhaustion (Cherry et al., 2014; Serpas et al., 2023), and fatigue (Serpas et al., 2023). The present study findings suggest that aging adults with FM show greater hallmarks of physical frailty across distinct objective and self-reported indicators compared to their non-FM counterparts. However, the duration of FM symptoms was not independently associated with performance-based or self-reported frailty, suggesting that frailty risk may be more closely related to current symptom severity than cumulative symptom exposure. Of note, this observation should be interpreted cautiously. Although analyses were adequately powered, the null association between symptom duration and frailty may nonetheless reflect measurement-related factors such as recall bias of self-reported symptoms or imprecision in estimating FM onset. In addition, duration alone may be insufficient to capture the cumulative burden of pathology, as this metric does not capture symptom intensity, treatment history, or periods of remission. Prospective longitudinal designs are needed to more precisely estimate the relative contributions of current symptom severity versus cumulative exposure.

Extending FM frailty research would benefit the empirical progress of establishing reliable and valid metrics of frailty for the FM population and would help to identify clinically relevant characteristics critical in the clinical conceptualization and treatment of frailty in FM. Additional FM characteristics and symptoms are likely relevant in the onset, degree, and severity of frailty in FM and should be explored in future research.

The present study used physical frailty domains based on the physical frailty phenotype identified by Fried et al. (2001) and Xue (2011). These included weakness, low physical activity, exhaustion, and slowness. BMI was included as a covariate in our study rather than the fifth domain of weight loss. A recent study (Wysham et al., 2025) used the FRAIL scale to define frailty across multiple rheumatic conditions (e.g., rheumatoid arthritis, osteoarthritis, FM, connective tissue diseases). This scale incorporated fatigue, resistance (difficulty climbing 10 steps), ambulation, illness (≥5 comorbidities), and weight loss. Note that traditionally these domains have been assessed with self-report questions. The current study incorporated both performance-based and self-report measures and found that 45% of the variance in frailty was explained by objective measures (FAB, 6MWT, 30SCS, 8FUPGT, and 30FW), whereas only 15% of the variance was explained by self-report. These physical performance measures assessed balance, aerobic endurance, lower body strength, functional mobility, and gait velocity, respectively. Lee et al. (2025) also used objective measures of gait speed and hand-grip strength in addition to self-report measures of physical activity level, exhaustion, and weight loss to assess frailty with participants (n = 373) across four different memory clinics. Physical assessments were well-received by patients, caregivers, and healthcare providers.

The cross-sectional nature of this study provides an opportunity for future research to investigate trajectories of frailty across the lifespan potentially with intensive longitudinal designs or experience sampling strategies. This would clarify the pathophysiology of frailty in aging adults with FM. Furthermore, the state of research on frailty and FM highlights significant knowledge gaps in identifying the prevalence, incidence, and mechanisms of frailty among aging adults with FM. For instance, research has shown that sleep quality mediates the association between chronic pain and frailty among Chinese patients with cancer (Zhang et al., 2023), and poorer sleep quality has been associated with worse objectively measured postural control in FM (Serpas et al., 2024). Therefore, future research is needed to evaluate and estimate frailty and its mechanisms in FM potentially through a feasible and cost-effective combination of objective and self-report indicators of frailty as utilized in this study to empirically evaluate frailty phenotypes.

This study conceptualized physical frailty based on previous research (Fried et al., 2001; Xue, 2011). Several methods of conceptualizing and assessing frailty exist with a mixture of objective and self-report assessments across various domains beyond solely physical frailty (Faller et al., 2019). This study provided an assessment of largely objective physical-based frailty among FM participants compared to pain-free controls. Future research is needed to examine additional phenotypes of frailty (Faller et al., 2019). Moreover, the generalizability of these findings is limited by the demographic composition of the sample, which was predominantly white, female, and mid-high socioeconomic status. Thus, the study results may not extend to more sociodemographically or socioeconomically diverse populations, men, or people of color who may experience different patterns of pain, healthcare access, and physical frailty risk. These sample limitations identify opportunities for future research. For instance, since frailty is more common among people of color (Bandeen-Roche et al., 2015), tailoring frailty-based measurement and recruitment strategies and interventions to address population needs should be considered. Relatedly, recent evidence suggests that social frailty, defined by reduced social participation, isolation, and limited access to supportive resources, independently predicts well-being in older adults (Coundouris et al., 2025). Integrating assessments of physical and social frailty may be particularly important for individuals with disabilities, for whom stigma and social exclusion represent chronic psychosocial stressors that may accelerate functional decline (Serpas et al., 2025). Lastly, a recent systematic review of 39 studies indicated that comorbid depression and pain intensity were identified as common barriers to physical activity in FM (Vancampfort et al., 2023). Considering that physical activity is associated with reduced risk of frailty (Zhao et al., 2022), existing interventions aimed at enhancing functioning in prefrail and frail adults should tailor interventions to target pain (e.g., Kidd et al., 2019; Puts et al., 2017). Thus, future research should focus on identifying and evaluating effective strategies to enhance physical activity uptake in the context of frailty management for aging adults with FM.

## 5. Conclusions

This study is, to our knowledge, the first to investigate differences in frailty between aging adults with or without FM through novel operationalization of physical frailty using both objective and self-report indicators. This study confirmed our hypothesis that aging adults with more pain (FM participants) demonstrated greater physical frailty than their non-FM counterparts. However, contrary to predictions, the length of time since FM symptom onset was not significantly associated with markers of physical frailty. Thus, variability in physical frailty among aging adults with FM does not appear to be associated with time since symptom onset.

## Figures and Tables

**Table 1:** Descriptive data of sociodemographic and frailty variables of the study sample (n = 234).

Variable	M (SD)	n (%)
Age (years)	63.79 (8.72)	-
Pain (score 0–10)	4.38 (2.95)	-
Fibromyalgia	5.98 (2.30)	
Non-fibromyalgia	2.07 (2.13)	
BDI-II	11.56 (9.66)	-
BMI (kg/m^2^)	28.11 (5.86)	-
6MWT (yards)	561.15 (117.70)	-
FAB (score 0–40)	32.34 (5.00)	-
30SCS (number of stands)	11.71 (3.86)	-
8FUPGT (seconds)	5.84 (1.42)	-
30FW (ft/s)	2.38 (0.34)	-
RAPA 1 (score 1–5)	3.98 (1.20)	-
RAPA 2 (score 1–3)	2.08 (0.82)	-
Fatigue (score 0–10)	4.61 (3.00)	
Fibromyalgia status		
Yes	-	138 (59.0%)
Gender		
Female	-	195 (83.3%)
Race/ethnicity ^†^		
White	-	90 (89.1%)
Hispanic	-	14 (14.5%)
African American	-	2 (2.0%)
Asian American	-	5 (2.1%)
Multiracial	-	1 (1.0%)
American Indian or Alaska Native	-	2 (2.0%)
Other	-	4 (4.0%)
Income		
<USD 9000	-	2 (0.9%)
USD 10,000–29,999	-	30 (12.9%)
USD 30,000–59,999	-	61 (26.1%)
USD 60,000–89,999	-	48 (20.5%)
USD 90,000–199,999	-	63 (26.9%)
USD 200,000+	-	10 (4.3%)
Employment status		
Retired	-	104 (44.8%)
Working full time	-	36 (15.5%)
Working part time	-	28 (12.1%)
Looking for work	-	7 (3.0%)
Keeping house	-	18 (7.8%)
Unemployed/on leave/disabled	-	32 (13.8%)
Other	-	7 (3.0%)
Marital status		
Never married	-	3 (2.9%)
Married	-	63 (61.2%)
Divorced/separated	-	25 (24.3%)
Widowed	-	12 (11.7%)
Highest completed education		
High school or less	-	12 (6.0%)
Some college/trade school	-	71 (30.3%)
College degree	-	72 (30.8%)
Professional/graduate degree	-	78 (33.3%)

Note. n = 234. BDI-II = Beck Depression Inventory-II; BMI = body mass index; 6MWT = 6-minute walk test; FAB = Fullerton Advanced Balance; 30SCS = 30-second chair stand; 8FUPGT = 8-foot up and go test; 30FW = 30-foot walk; RAPA = Rapid Assessment of Physical Activity. ^†^ Percentages may exceed 100 because participants were allowed to select more than one category.

**Table 2:** Bivariate correlations among primary study variables.

	1	2	3	4	5	6	7	8	9	10	11	12
1. Age	-											
2. Pain	−0.350 ***	-										
3. BDI-II	−0.319 ***	0.596 ***	-									
4. BMI	−0.069	0.240 ***	0.176 **	-								
5. 6MWT	−0.165 *	−0.397 ***	−0.400 ***	−0.369 ***	-							
6. FAB	−0.303 **	−0.248 ***	−0.323 ***	−0.234 ***	0.573 ***	-						
7. 30SCS	0.021	−0.415 ***	−0.422 ***	−0.237 ***	0.547 ***	0.467 ***	-					
8. 8FUPGT	0.222 ***	0.200 ***	0.185 **	0.267 ***	−0.611 ***	−0.564 ***	−0.636 ***	-				
9. 30FW	−0.006	−0.216 ***	−0.200 **	−0.063	0.360 ***	0.274 ***	0.404 ***	−0.413 ***	-			
10. RAPA 1	0.144 *	−0.351 ***	−0.419 ***	−0.254 ***	0.350 ***	0.312 ***	0.345 ***	−0.296 ***	0.176 **	-		
11. RAPA 2	0.098	−0.252 ***	−0.308 ***	−0.194 **	0.266 ***	0.189 ***	0.343 ***	−0.208 **	0.193 **	0.531 ***	-	
12. Fatigue	−0.314 ***	0.732 ***	0.638 ***	0.179 **	−0.383 ***	−0.275 ***	−0.391 ***	0.224 ***	−0.172 **	−0.377 ***	−0.326 ***	-

Note. n = 234. BDI-II = Beck Depression Inventory-II; BMI = Body Mass Index; 6MWT = 6-Minute Walk Test; FAB = Fullerton Advanced Balance; 30SCS = 30-Second Chair Stand; 8FUPGT = 8-Foot Up and Go Test; 30FW = 30-Foot Walk; RAPA = Rapid Assessment of Physical Activity. * *p* < 0.05, ** *p* < 0.01, *** *p* < 0.001.

**Table 3:** Results from principal component analysis with oblique rotation of performance-based and self-reported frailty measures.

Measure	*h* ^2^	Rotated Component	
		Objective-Based Frailty	Self-Reported Frailty
8FUPGT	0.74	**−0.89**	0.08
6MWT	0.67	**0.76**	0.12
FAB	0.57	**0.76**	0.01
30SCS	0.65	**0.71**	0.19
30FW	0.38	**0.64**	−0.07
RAPA2	0.69	−0.10	**0.86**
RAPA1	0.68	0.03	**0.82**
Fatigue	0.47	−0.13	**−0.62**
Total Variance Explained:	60.59%		

Note. Results from the rotated pattern matrix are presented with loadings sorted by size. 8FUPGT = 8-Foot Up and Go Test; 6MWT = 6-Minute walk test; 30FW = 30-Foot Walk; FAB = Fullerton Advanced Balance; 30SCS = 30-Second Chair Stand; RAPA = Rapid Assessment of Physical Activity. Values are bolded to denote salient loadings and to illustrate the simple structure of the solution.

## Data Availability

The data presented in this study are not publicly available due to privacy and ethical restrictions. However, they may be available from the corresponding author upon reasonable request, provided that appropriate ethical approval has been obtained.

## Abbreviations

The following abbreviations are used in this manuscript:

FM	Fibromyalgia
MMSE	Mini Mental Status Exam
SPSS	Statistical Package for the Social Sciences
PCA	Principal component analysis
KMO	Kaiser–Meyer–Olkin
MCAR	Missing completely at random
BDI-II	Beck Depression Inventory-II
6MWT	6-minute walk test
8FUPGT	8-foot up and go test
FAB	Fullerton Advanced Balance
30SCS	30-second chair stand
30FW	30-foot walk
RAPA	Rapid Assessment of Physical Activity
ANOVA	Analysis of variance
